# Preventing Weight Gain in Women in Rural Communities: A Cluster Randomised Controlled Trial

**DOI:** 10.1371/journal.pmed.1001941

**Published:** 2016-01-19

**Authors:** Catherine Lombard, Cheryce Harrison, Samantha Kozica, Sophia Zoungas, Sanjeeva Ranasinha, Helena Teede

**Affiliations:** 1 Monash Centre for Health Research and Implementation, Monash University, Melbourne, Australia; 2 Department of Nutrition and Dietetics, Monash University, Melbourne, Australia; 3 Diabetes and Vascular Medicine Unit, Monash Health, Melbourne, Australia; Massachusetts General Hospital, UNITED STATES

## Abstract

**Background:**

Obesity is reaching epidemic proportions in both developed and developing countries. Even modest weight gain increases the risk for chronic illness, yet evidence-based interventions to prevent weight gain are rare. This trial will determine if a simple low-intensity intervention can prevent weight gain in women compared to general health information.

**Methods and Findings:**

We conducted a 1-yr pragmatic, cluster randomised controlled trial in 41 Australian towns (clusters) randomised using a computer-generated randomisation list for intervention (*n* = 21) or control (*n* = 20). Women aged 18 to 50 yr were recruited from the general population to receive a 1-yr self-management lifestyle intervention (HeLP-her) consisting of one group session, monthly SMS text messages, one phone coaching session, and a program manual, or to a control group receiving one general women’s health education session. From October 2012 to April 2014 we studied 649 women, mean age 39.6 yr (+/− SD 6.7) and BMI of 28.8 kg/m^2^ (+/− SD 6.9) with the primary outcome weight change between groups at 1 yr. The mean change in the control was +0.44 kg (95% CI −0.09 to 0.97) and in the intervention group −0.48kg (95% CI −0.99 to 0.03) with an unadjusted between group difference of −0.92 kg (95% CI −1.67 to −0.16) or −0.87 kg (95% CI −1.62 to −0.13) adjusted for baseline values and clustering. Secondary outcomes included improved diet quality and greater self-management behaviours. The intervention appeared to be equally efficacious across all age, BMI, income, and education subgroups. Loss to follow-up included 23.8% in the intervention group and 21.8% in the control group and was within the anticipated range. Limitations include lack of sensitive tools to measure the small changes to energy intake and physical activity. Those who gained weight may have been less inclined to return for 1 yr weight measures.

**Conclusions:**

A low intensity lifestyle program can prevent the persistent weight gain observed in women. Key features included community integration, nonprescriptive simple health messages, small changes to behaviour, low participant burden, self-weighing, and delivery including a mix of group, phone, and SMS text reminders. The findings support population strategies to halt the rise in obesity prevalence.

## Abstract 2

In a pragmatic, cluster-randomised controlled trial, Catherine Lombard and colleagues assess the value of a self-management lifestyle intervention to prevent weight gain among women living in rural Australia.

## Editors' Summary

**Background:**

Obesity—having an unhealthy amount of body fat—is a global public health problem. In the US, for example, more than one-third of adults are obese and another third are overweight. Obesity is defined as having a body mass index (BMI; an indicator of body fat calculated by dividing a person’s weight in kilograms by their height in meters squared) of equal to or more than 30 kg/m^2^; overweight individuals have a BMI of 25.0–29.9 kg/m^2^. Increased body fat is associated with an increased risk of developing diabetes, cancer, cardiovascular disease and other chronic diseases.. People gain body fat by consuming food and drink that contains more energy (calories) than they need for their daily activities. So excess body fat can be prevented and reversed by eating a diet that contains fewer calories and by being more active.

**Why Was This Study Done?:**

BMI increases with age in most adults although in recent years young adults have been shown to be gaining body fat faster than older adults. However, the adult weight gain per year is generally less than 1 kg and could be prevented by encouraging people to eat just a little less and exercise just a little more. Prevention of weight gain is likely to be easier than reversal of established obesity, but few interventions designed to prevent weight gain have been rigorously tested. In this pragmatic randomized controlled trial, the researchers investigate whether a simple low-intensity intervention can prevent weight gain among 18–50-year-old women living in rural communities in Australia. Rates of obesity are generally higher among women than men and, in affluent countries, rural-dwelling women have higher rates of weight gain and obesity than urban-dwelling women—in Australia, young women living in rural and metropolitan areas gain an average of 700 g and 550 g per year, respectively. A pragmatic cluster randomized controlled trial randomly assigns groups of people (here, women living in different towns) to receive alternative interventions and compares outcomes in the differently treated “clusters” under real-life conditions.

**What Did the Researchers Do and Find?:**

The researchers assigned 41 Australian towns to receive a 1 yr self-management lifestyle intervention (HeLP-her) or to act as controls. The intervention consisted of one group session during which facilitators delivered general health information and five simple health messages (for example, try to eat two servings of fruit and five servings of vegetables a day), a program manual to help participants develop a personalized weight gain prevention strategy, monthly text message to remind participants of key behaviors for weight gain prevention, and a 20-min personal phone coaching session delivered three months into the trial. Participants in the control clusters received a group education session on general women’s health topics at the start of the trial. In total, 649 women with an average baseline BMI of 28.2kg/m^2^ participated in the trial. After one year, the average weight change was +0.44 kg in the control arm of the trial and −0.48 kg in the intervention arm (a between group difference in weight change of −0.92 kg). The intervention also improved diet quality and self-management behavior and was equally efficacious across all age, BMI, income, and education subgroups.

**What Do These Findings Mean?:**

These findings suggest that a low-intensity lifestyle program can prevent persistent weight gain among women. Specifically, the year-long HeLP-her intervention prevented a weight gain of nearly 1 kg on average among women living in rural Australia. Notably, a recent modeling study estimated that a 1 kg weight loss, if applied across the US population, could avoid 2 million cases of diabetes, 1.5 million cases of cardiovascular disease, and more than 73,000 cases of cancer. Although it is difficult to identify the successful elements of any intervention that targets multiple behaviors, key components of the HeLP-her intervention probably include the use of simple, non-prescriptive health messages, the focus on small behavioral changes, regular self-weighing, and the use of both personal and electronic means to deliver the intervention. Some aspects of this trial (for example, nearly a quarter of the participants did not complete the trial) may affect the accuracy of its findings and a longer follow-up is needed to determine the long-term effects of the intervention. Nevertheless, these findings provide new information on effective weight gain prevention strategies that align with current clinical guidelines and population strategies designed to halt the global rise in obesity.

**Additional Information:**

This list of resources contains links that can be accessed when viewing the PDF on a device or via the online version of the article at http://dx.doi.org/10.1371/journal.pmed.1001941.

- The World Health Organization provides information on obesity (in several languages)
- The Global Burden of Disease website provides the latest details about global obesity trends; the International Obesity Taskforce also provides information about the global obesity epidemic
- The UK National Health Service Choices website provides information about obesity (including some real stories), healthy eating, exercising
- The US Centers for Disease Control and Prevention has information on all aspects of overweight and obesity
- ChooseMyPlate.gov is a resource provided by the US Department of Agriculture that provides individuals and health care professionals with user-friendly information on nutritional and physical exercise
- The US National Institute of Diabetes and Digestive and Kidney Diseases provides information on weight control and healthy living
- MedlinePlus provides links to other sources of information on obesity (in English and Spanish)
- More information about obesity in Australia, this trial, and the HeLP-her intervention is available

## Introduction

Obesity is a complex public health problem affecting almost all age and socioeconomic groups. Rates of obesity are increasing in both developed and developing countries with higher rates reported for women than men [1]. In Australia, the United Kingdom, and the United States, just over 30% of adults are considered a healthy weight, and projections based on current trends indicate that there will be 65 million more obese adults in the US and 11 million more obese adults in the UK by 2030 [2]. Because obesity is now occurring at a younger age, a greater proportion of the population will be living with obesity-related chronic illness for longer than previous generations [3]. Body mass index (BMI) increases progressively in most adults, and the mean annual weight gain, estimated to be less than 1 kg per yr, could be prevented with small changes to energy balance [4]. In support of prevention of weight gain, WHO has introduced a target to halt the rise in obesity prevalence by 2025 [5].

Obesity, once established, is difficult to treat with clinical service providers and public health organisations increasingly dealing with complex and costly obesity-related health problems. Clinical obesity guidelines urge health care practitioners to deliver intensive, prescriptive interventions and to monitor and support weight gain prevention [6]. Increasing demands on practitioners’ time and limited success in weight loss treatments suggests a target to prevent weight gain is a more realistic option, applicable to all population groups with potential for higher patient satisfaction, commitment, and success.

Evidence-based interventions to prevent weight gain are rare and heterogeneous, with equivocal results [7]. Just four trials combining physical activity and healthy eating specifically aimed at the prevention of weight gain have been identified [8]. The United States Agency for Healthcare Research and Quality has concluded the evidence for weight gain prevention is based on very few studies of mixed quality, and there is need for further methodologically rigorous research [9]. Health care providers in both clinical and public health settings have little effective empirical models to guide practice. Evidence-based weight gain prevention interventions that are easy to deliver are urgently needed.

Reproductive-aged women are an important target group with longitudinal studies showing rapid weight gain [10] and barriers to participation in obesity-protective behaviours. In developed countries, rural-dwelling women are further disadvantaged with higher rates of weight gain and obesity [11]. In Australia, the proportion of females who are overweight or obese increases by 22% between ages 25 and 54, compared to a 15% increase in prevalence in similarly aged males [12]. Australian longitudinal data suggests weight gain is persistent in women across all BMI categories but is greatest in young rural women who gain 700 g/yr (self-reported), compared to 550 g/yr in metropolitan dwelling women [10]. In addition to increased risk for chronic illness, reproductive-aged overweight women also carry high risks of adverse maternal and foetal outcomes [13]. Women “model” health behaviours for their children [14]; therefore, targeting women has potential to increase the reach of preventative interventions.

The primary aim of this pragmatic community-integrated, cluster randomised controlled trial (RCT) is to determine if a simple, low-intensity, self-management lifestyle intervention (HeLP-her) can prevent weight gain in young to middle-aged women. We hypothesised that a rural-based population of women who participated in the intervention would gain less weight over one year than a control group assigned to receive generic health information.

## Methods

### Population, Randomisation, and Masking

Victoria is a state of Australia with a population of 5.71 million and is divided geographically into 34 local government shires. Twelve shires were excluded because of potential confounding due to participation in Healthy Together Victoria, a government preventative health campaign. All rural towns with populations between 2,000 and 10,000 within the remaining 22 shires and located within a radius of 100 and 400 km from the state capital were eligible for randomisation. Forty-four towns met these criteria, and 42 towns were randomised by the study biostatistician using a computer-generated randomisation list. Randomisation occurred at the town (cluster) level and analysis at the individual level. One town was withdrawn because of intensive seasonal farming practices (harvesting) limiting recruitment opportunities. Clustering by town eliminated potential contamination which may have occurred between participants living in the same small communities.

**Fig 1:**
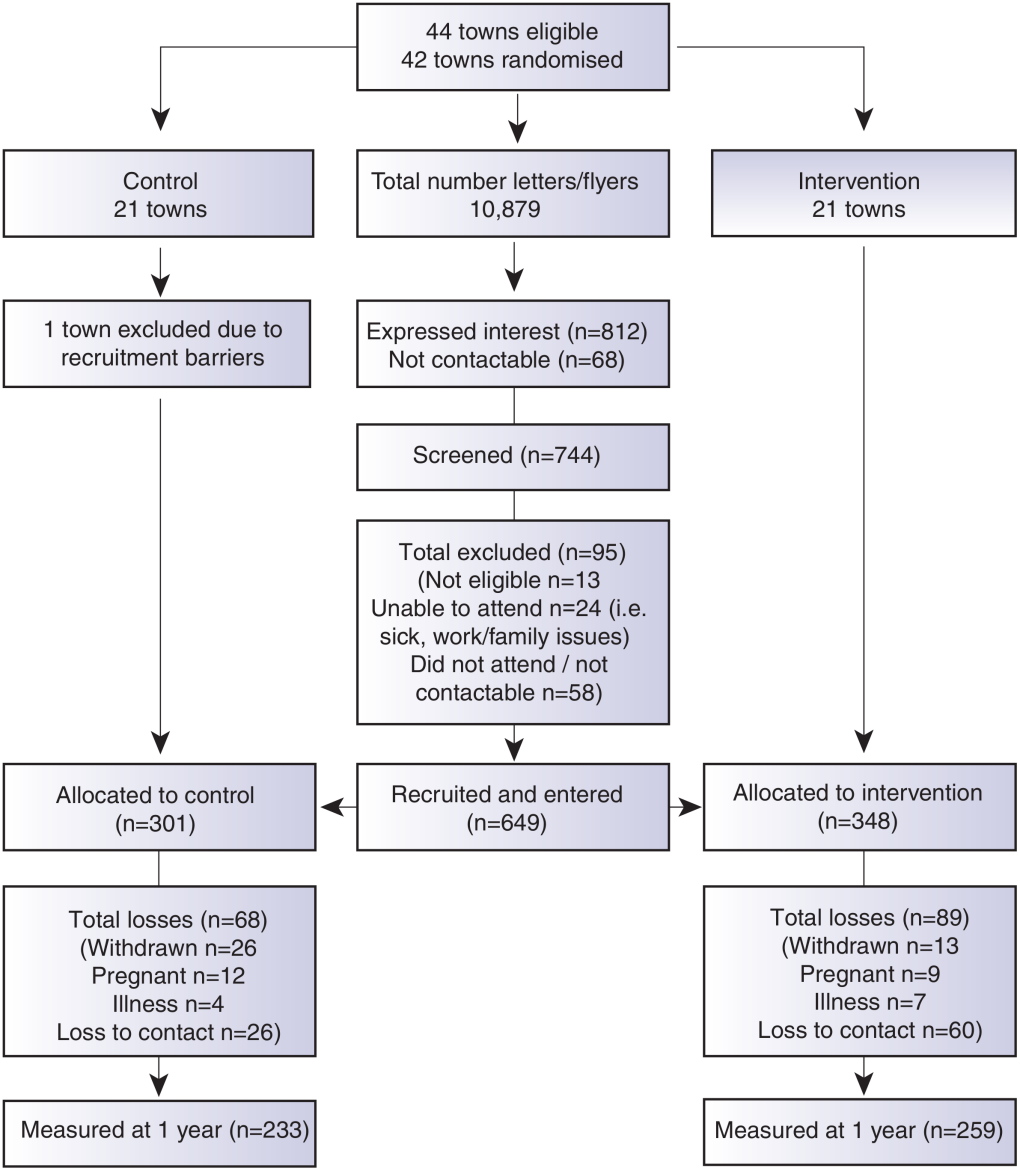
CONSORT diagram describing the flow of participants through the study.

A comprehensive communication plan and engagement framework was developed to ensure multilevel community integration, and is described elsewhere [15]. Participants were recruited as clusters according to the town of residence between September 2012 and April 2013. Recruitment occurred by a letter of invitation and flyers distributed in the general community through primary schools, preschools, health clinics, clubs, retail businesses, supermarkets, and childcare centres in each town. Research staff also personally recruited participants in the main retail areas in each town and placed advertisements in local newspapers. Screening occurred by phone or in person, and eligibility criteria included female, age of 18–50 yr, and residing in or near participating towns. Eligible participants signed informed consent prior to commencement of the first session. To be as inclusive of the population as possible, all BMI categories were included. Exclusion criteria were minimal and included pregnancy or serious medical conditions that would inhibit full participation in the program. Further detail is described in the trial protocol [15] and the CONSORT diagram (Fig 1).

Due to the nature of the trial, researchers were aware of group allocation at baseline. Participants were not aware of group assignment, although they were aware that they were participating in a healthy lifestyle research program. At the 1 yr data collection point, both participants and new field researchers were blinded to group allocation and previous anthropometric measures. Ethics approval was granted by Monash Health Human Research Ethics Committee, all participants signed consent prior to participation. The trial was registered with the Australian New Zealand Clinical Trials Register (12612000115831) prior to recruitment.

### Intervention

The intervention (HeLP-her) was based on the self-determination and cognitive behavioural theory, with motivational interviewing the primary method of interaction with participants. Behavioural strategies were informed by established practices [16]. Participants attended one facilitator-led interactive small group session held in each town. The trained facilitator presented lifestyle information related to weight gain in the form of five simple health messages (e.g., try to eat two servings of fruit and five servings of vegetables per day; take a brisk walk for at least 30 min on most days of the week). Using the topics and activities in the program manual as a guide, participants identified personal health priorities and practiced skills in goal setting, problem solving, relapse prevention, and self-monitoring. Participants were assisted by the facilitator to generate goals and action plans based on their personal priorities. Each participant therefore developed a personalised weight gain prevention strategy. Participants were instructed to work through the manual over the next four weeks in their own time. Intervention participants received an SMS text message every 4 wk to reinforce program messages. At 12 wk, they participated in one 20-min phone coaching session, delivered by staff trained in motivational interviewing, which utilised client-orientated counselling to explore and resolve ambivalence and review progress (Box 1).

**Box 1. Intervention components**

**Community engagement**

Regional government departments and community and school leaders were contacted by email and a follow-up phone call. They were invited to support the program implementation by providing introductions to key community groups, assistance with recruitment, and providing facilities for program delivery. Information on local programs that could support the intervention was also obtained.

**Group session**

One 60-min group session was held with 8–15 women at community locations such as schools or halls. Facilitators delivered general health information plus simple health messages.

Facilitators using an interactive model and supported by the program manual, worked through examples of behavioural self-management skills including setting health priorities, problem solving, and self-monitoring, focusing on small changes to behaviour.

**Program manual**

The manual included information to increase knowledge, personal stories and activities to challenge personal beliefs and behaviours and opportunities to develop self-management skills such as problem solving and action planning. Participants completed initial activities in the interactive group session and worked through manual activities in their own time.

**Phone coaching**

One 20-min phone coaching session at week 12 was delivered by trained coaches and aimed to assist completion of manual activities and to reinforce intervention messages and generate action plans.

**SMS text messages and support**

From week 4, one text message was sent every 4 wk consistent with program messages to remind participants of key behaviours. Text messages were personalised by name only and were otherwise generic.

### Control

The control group received one 45-min group education session on general women’s health topics including the readily available Australian Dietary Guidelines [17] and the Australian Physical Activity Guidelines [18]. The session was not interactive, and women were not given any individual advice. No further contact with the control group occurred until the follow up visit at 1 yr.

Trained facilitators delivered the intervention and control group sessions. To ensure high program fidelity, we centralised training and used standardised delivery methods and resources. In addition, working in pairs, a facilitator delivered the intervention and a field researcher observed each session and completed a program checklist. Participants were provided with the study contact details to enable spontaneous reporting of any adverse events.

### Outcome Measures

Anthropometric measures (measured weight, height, waist, and hip) and self-completed questionnaires were collected prior to intervention commencement (September 2012 and April 2013) and at 1 yr (September 2013 to April 2014). Weight was objectively measured by a trained research assistant, using calibrated digital scales (Tanita WB110AZ) at baseline and 1 yr, with participants in light clothing, with an empty bladder, and without shoes. Waist and hip were measured in person twice with a nonelastic tape measure and a mean calculated. Height was measured at baseline only using a portable stadiometer. Questionnaires included demographic characteristics, health status, the international physical activity questionnaire (IPAQ) long form [19], and the Cancer Council Australia Food Frequency Questionnaire (FFQ) [20]. Self-management was assessed on a Likert scale using an adapted tool by Saelens [21] and included questions such as “I actively seek information about nutrition,” “I have food available for quick healthy meals,” and “I make back-up plans to make sure I get my physical activity.” Self-weighing was measured via a question with options including daily, weekly, monthly, occasionally, or never. Program satisfaction was measured by questionnaire at 1 yr on a Likert scale of 1–5, with higher scores indicating greater satisfaction.

### Statistical Analysis

The primary outcome, a priori, was weight gain at 1 yr. Sample size calculations estimated 196 participants were required for each group to detect at least a 1.0 kg difference in weight between groups based on the average annual weight gain of 0.8 kg in young Australian rural women and hypothesized a 0.2 kg reduction in weight as a result of the intervention (sd 3.5). We adjusted for the cluster design of the study, applying an intracluster correlation of 0.02 based on a previous trial in women [22]. Adjusting for the cluster design with a variance inflation factor (VIF) = 1.28, cluster size of 15, and allowing for 20% attrition over 1 yr, 600 women in 40 clusters of 15 women were to be recruited. To allow for inadvertent recruitment challenges, 42 towns (clusters) were randomised. The trial was designed to have a statistical power of 80%, with the use of a two-sided test at a significance level of 0.05.

All analyses were conducted according to the intention-to-treat principle. Baseline and within-group differences over time were assessed using paired Student’s *t* tests for continuous variables and χ^2^ tests for categorical variables. The effects of the intervention on study outcomes (between group differences) at 1 yr were analysed using linear regression with the variable of interest at 1 yr as the outcome variable, adjusted for baseline values, and obtained robust standard errors to adjust for the clustering effect of town in the regression models (the Huber/White/sandwich estimate of variance). In the first step, analyses were performed on the complete data. In the second step, analyses were performed on data that included values imputed using linear regression, multiple imputation with boot strapping. Effect modification by prespecified subgroups such as age (<30, 30–45, >45 yr), BMI (<25, 25–29.9, ≥30 kg/m^2^) country of birth (Australian born, overseas born), annual income (AUD <$40,000 $40,000–$80,000, >$80,000) highest qualification (no post-school certification, certificate/apprenticeship, diploma/bachelor’s degree), work status (full time, part-time, no paid work) and intervention group, on the primary outcome, (the difference in weight gain between baseline and 1 yr), was assessed using linear regression adjusted for clustering. Changes in dietary intake, leisure time physical activity, sitting time, and self-management behaviours in relation to the intervention health messages were also examined in subgroup analyses using linear regression adjusted for clustering.

## Results

### Participants

We recruited 649 women, mean age of 39.6 (+/− SD 6.7), from 41 randomised town clusters. The participants were equally represented across all BMI categories, 35.2% with a BMI ≤ 25 kg/m^2^, 31.9% with a BMI 25–29.9 kg/m^2^, and 33.0% with a BMI ≥ 30 kg/m^2^. Randomised towns were of low-to-moderate socioeconomic status. This is demonstrated by the socioeconomic index for areas (SEIFA) median score of 3 (on a scale of 1–10 deciles, where 1 represents greater disadvantage) for the randomised towns [23]. Participants were recruited from all income and education levels. There were no differences in baseline characteristics between the intervention and control groups, except for a minor age difference (Table 1). At 1 yr, weight was recorded for 76.2% of the intervention and 78.2% of the control group. Overall, 4.7% withdrew due to pregnancy or illness unrelated to the study (Fig 1). No baseline differences were observed between those who had weight recorded at 1 yr and those lost to follow-up (Table 2). Of the 22.2% of participants with missing weight data at 12 mo, 9.2% had a baseline BMI ≥ 30 kg/m^2^, 6.8% a BMI 25–29.9 kg/m^2^, and 6.2% a BMI of 18–24.9 kg/m^2^. No adverse events related to the trial were reported.

**Table 1:** Baseline characteristics.

Characteristic	Control * *n* = 301	Intervention* *n* = 348	*p*-Value
Age (yr) mean (SD)	39.0 (7.2)	40.1 (6.1)	0.02
Weight (kg) mean (SD)	78.0 (20.0)	78.7 (18.0)	0.54
BMI (kg/m^2^) mean (SD)	28.7 (6.7)	28.8 (6.5)	0.66
Waist circumference (cm) mean (SD)	94.5 (16.3)	95.7 (15.5)	0.29
Hip circumference (cm) mean (SD)	108.8 (13.9)	109.4 (12.5)	0.55
Height (cm) mean (SD)	165.0 (6.3)	165.5 (6.2)	0.39
Energy intake (kJ/day) mean (SD)	7,120 (2,887)	7,300 (3,769)	0.62
Energy expenditure, leisure time activity *MET min /week, mean (SD)	973 (1,577)	994 (1,925)	0.89
Income n (%)			
$40,000 or less	60 (22.9)	64 (22.3)	0.89
$41,000–$80,000	56 (21.3)	57 (19.9)	
More than $81,000	147 (55.9)	166 (57.8)	
Occupation *n* (%)			
Full-time paid work	47 (17.4)	59 (19.4)	0.42
Part-time	156 (56.9)	156 (51.6)	
No paid work	70 (25.7)	87 (28.8)	
Highest education *n* (%)			
No post-school qualifications	58 (21.4)	47 (15.5)	0.09
Certificate/Apprenticeship	83 (30.5)	85 (28.1)	
Diploma/Bachelor’s degree	131 (48.2)	171 (56.4)	
Born in Australia *n* (%)	255 (93.1)	283 (93.1)	0.10
Born outside Australia *n* (%)	19 (6.9)	21 (6.9)	

* Leisure time activity has been calculated using the leisure activity domain only of the IPAQ long version and expressed as MET-min calculated by multiplying the MET activity level (walking 3.3, moderate 4.0 and vigorous 8.0) by minutes performed each day.

**Table 2:** Baseline characteristics of participants with and without weight recorded at 1 year.

	Weight recorded at 1 yr, *n* = 492		Weight not recorded, *n* = 146		
	Intervention	Control	Intervention	Control	*p*-Value*
Weight (kg), mean (SD)	77.9 (18.1)	76.2 (18.8)	80.9 (17.7)	83.5 (23.0)	0.44
Age (yr), mean (SD)	40.7 (5.9)	39.6 (6.7)	37.7 (6.7)	36.1 (8.6)	0.26
BMI (kg/m^2^), mean (SD)	28.5 (6.4)	28.0 (6.5)	29.8 (6.5)	30.4 (7.6)	0.57
Waist (cm), mean (SD)	95.1 (15.2)	93.0 (15.2)	97.5 (15.6)	99.0(18.7)	0.58
Income AUD (%)					
$40,000 or less	22.0	18.4	23.3	41.2	0.11
$41,000 to $80,000	18.6	23.1	25.5	13.7	
More than $80,000	59.3	58.9	51.0	45.1	
Highest education %					
No post-school	14.9	22.4	18.2	17.0	0.38
Certificate/apprenticeship	24.2	29.7	45.4	34.0	
Diploma/Bachelor degree	60.9	48.0	36.6	49.1	
Occupation (%)					
Full time paid work	19.0	15.9	21.9	22.6	0.87
Part time	52.6	58.6	47.2	50.9	
No paid work	28.3	25.4	30.9	26.4	

*p-values represent no difference in baseline values between intervention and control participants who had missing weight at 12 mo.

### Weight Change

At 1 yr, the mean change in weight from baseline was +0.44 kg (95% CI, −0.09 to 0.97, *n* = 233) for the control group and −0.48 kg (95% CI, −0.99 to 0.03, *n* = 258) for the intervention group, with a mean unadjusted difference between the groups of −0.92 (−1.67 to −0.16) and −0.87 kg (95% CI, −1.62 to −0.13 *p* = 0.02) after adjusting for baseline values and the potential clustering effect by town (Table 3). Following multiple imputation to account for missing values, the mean difference between the groups remained significant (*p* = 0.02) (Table 3).

**Table 3:** Weight measures and difference between the intervention and control groups.

	Control Group *n* = 233	Intervention Group *n* = 259	Adjusted Difference^‡^ *n* = 492	Multiple Imputation *n* = 649	Intra Class Correlation (ICC)
**Weight (kg)**					
Baseline mean (SD)	76.16 (18.73)	77.99 (18.01)			0.11
Follow-up mean (SD)	76.60 (18.85)	77.51 (18.06)			
Change mean (95% CI) ^¥^	0.44 (−0.09 to 0.97)	−0.48 (−0.99 to 0.03)	−0.87 (−1.62 to −0.13)*	−0.71(−1.41 to −0.005)*	
**Waist circumference (cm)**					
Baseline mean (SD)	93.03 (15.20)	95.07 (15.26)			0.09
Follow-up mean (SD)	93.66 (15.43)	94.64 (15.86)			
Change mean (95% CI)	0.63 (−0.20 to 1.50)	−0.43 (−1.13 to 0.27)	−0.96 (−2.30 to 0.41)	−0.78 (−2.02 to 0.47)	
**Waist:hip ratio**					
Baseline mean (SD)	0.86 (0.06)	0.87 (0.07)			0.007
Follow-up mean (SD)	0.86 (0.07)	0.87 (0.07)			
Change mean (95% CI)	0.0012 (−0.005 to 0.008)	−0.0001 (− 0.006 to 0.006)	0.0007 (−0.007 to 0.009)	0.0004 (−0.008 to 0.009)	

* *p* < 0.05

^‡^ Linear regression was used to assess the difference between groups over the length of the study. All data were adjusted for town cluster and baseline value of interest.

^¥^ Change within groups assessed using paired Student’s *t* tests.

### Subgroup Analysis

**Fig 2:**
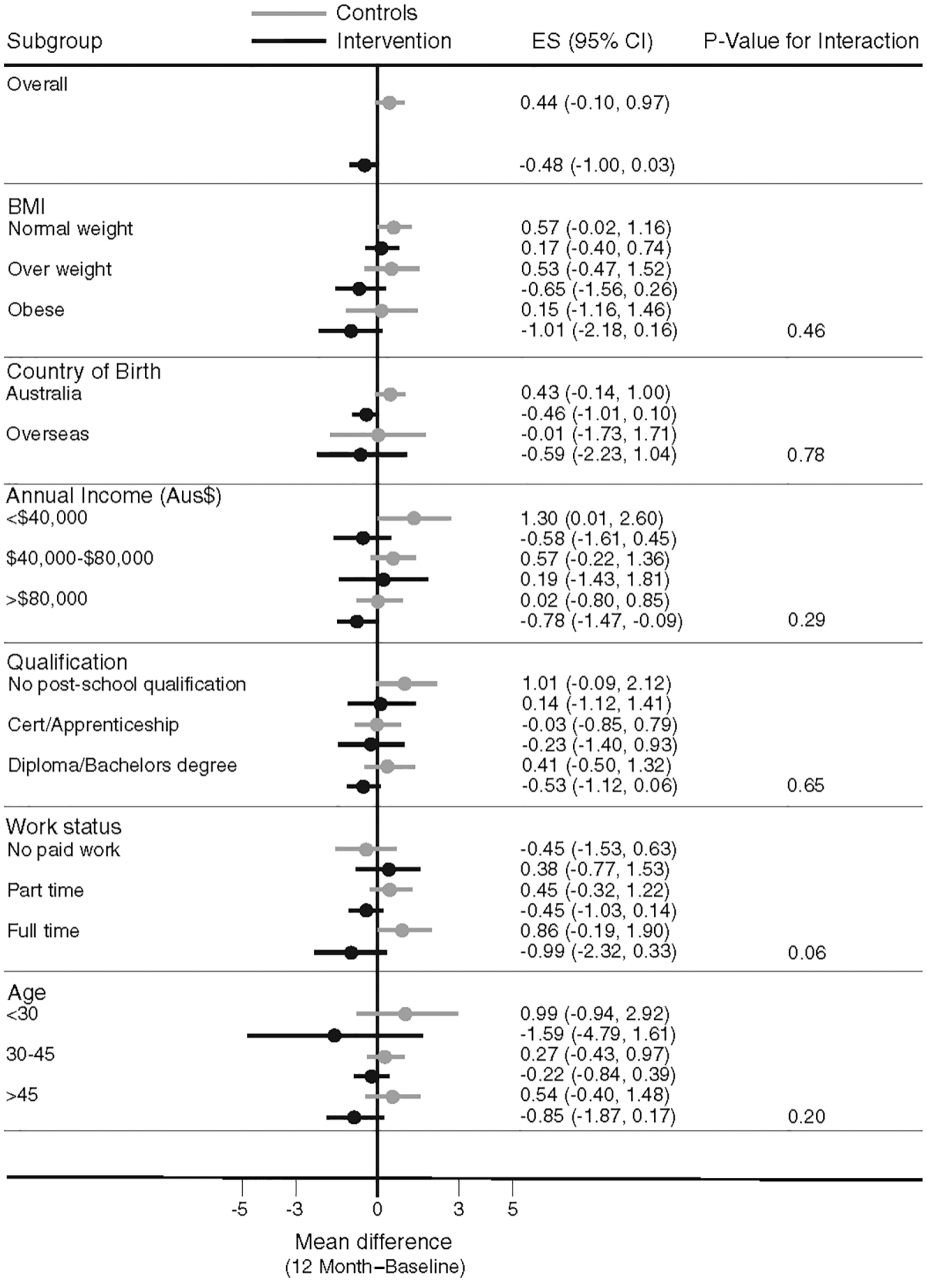
Mean difference in weight change by baseline subgroups.

**Fig 3:**
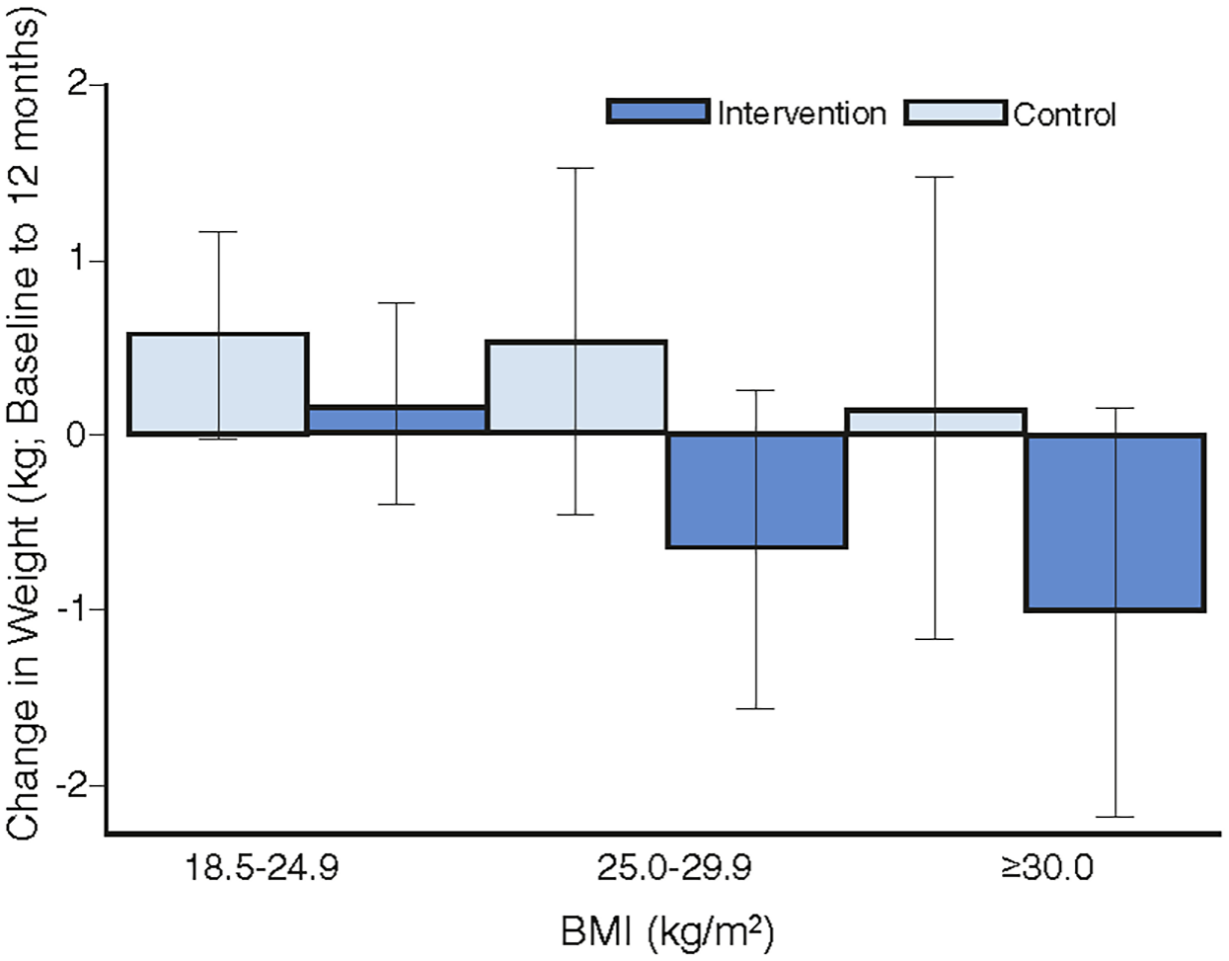
The mean difference in weight change by intervention group for BMI categories.

**Fig 4:**
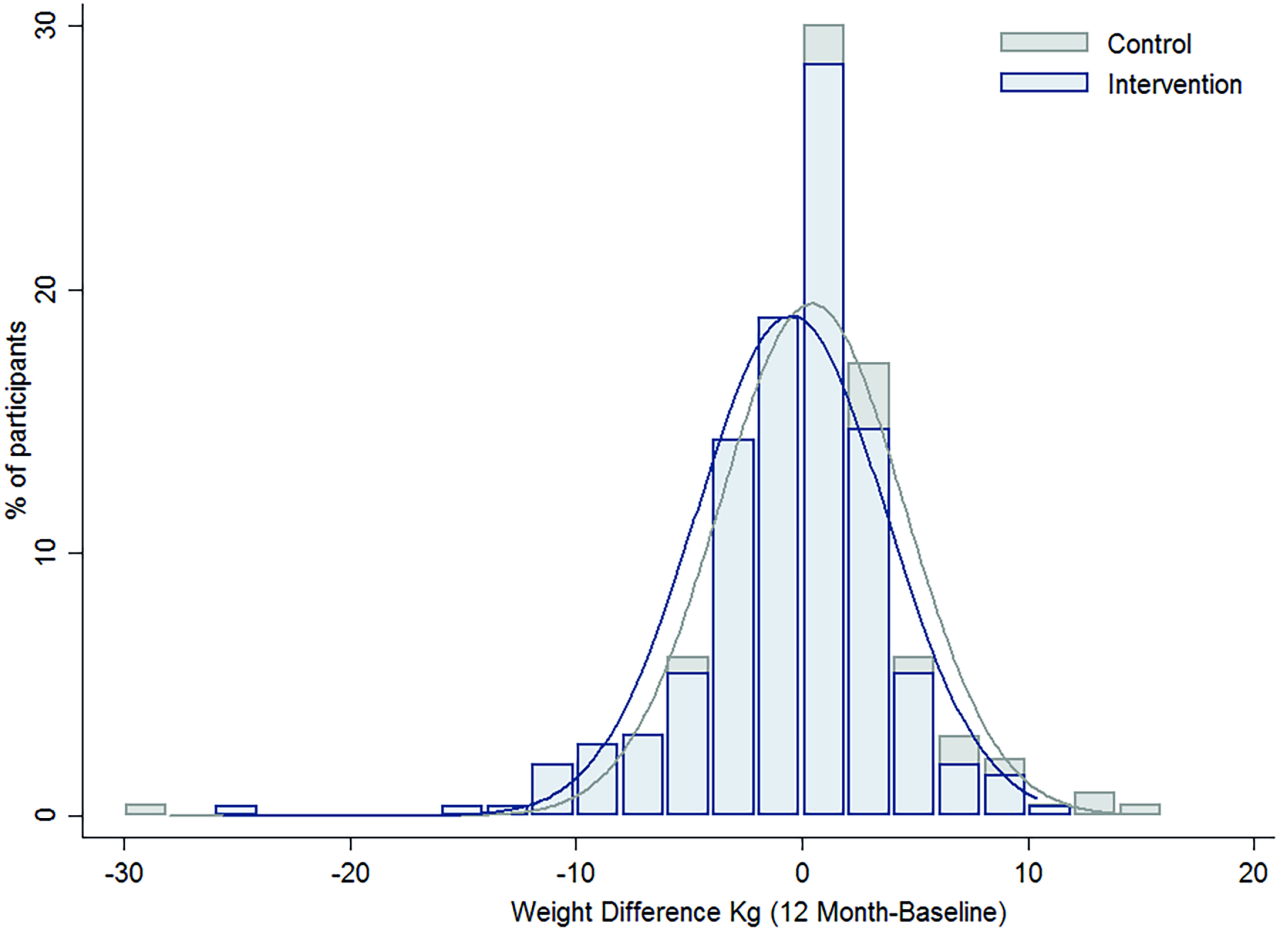
The difference in the distribution of weight gain over one year by intervention group.

Overall, there was no heterogeneity in the intervention effects by any baseline category examined (Fig 2), with the exception of work status where intervention participants in “no paid work” achieved a greater weight difference than other work categories. Of note, the largest weight gain occurred in the control group, BMI category 18 to 24.9 kg/m^2^ (0.57 kg, 95% CI, −0.02 to 1.16), while the largest weight loss was observed in the intervention participants with a BMI ≥ 30 kg/m^2^ (−1.01kg, 95% CI, −2.18 to 0.16), with the greatest weight difference observed in those with a BMI 25–29.9 kg/m^2^ (a mean difference of −1.18kg (95% CI, −2.6 to 0.21) (Fig 3). Greater weight loss occurred in intervention women who regularly self-weighed (−1.39 kg) compared to those that did not self-weigh (0.37 kg), a difference of −1.76 kg, (95% CI −2.35 to −0.44). When we compared the regular self-weighers in the intervention group with the control group, we observed a difference of −1.22 kg (95% CI –2.46 to 0.02) in favour of the intervention group, although this did not reach significance. There was a shift in the distribution of weight gain in favour of the intervention following participation in the program (Fig 4).

A further analysis of weight change from baseline to 12 mo by percentiles revealed the intervention group consistently lost more weight than the control group across all percentiles. The weight difference is more pronounced at the 5th and 25th percentiles for the intervention group (−9.0 kg and −2.4 kg) compared to the control group (−4.7 kg and −1.5 kg), respectively. The median weight difference in the intervention group was 0.1 kg compared to 0.5 kg for the controls. The Inter Quartile Range (IQR) for the intervention group was −2.4 to 1.8 compared to −1.5 to 2.5 for the control group.

### Lifestyle Behaviour Change

Both intervention and control groups reported a reduction in energy intake at 1 yr. The diet quality improved within the intervention group, who increased fruit intake and reduced snack food, takeaway food, and alcohol consumption, which matched intervention messages, suggesting good message uptake. There was less change observed in the control group as anticipated, and despite the changes in the intervention group, the between-group differences were not significant (Table 4). There was no change detected in leisure time physical activity or sitting time observed in either group at 1 yr. The intervention group reported an increase in self-management strategy use related to diet and physical activity at 1 yr with a significant difference in dietary self-management observed between the groups (Table 4).

**Table 4:** Mean change in diet and physical activity related behaviours over 1 yr.

Variable	Control *n* = 233	Intervention *n* = 259	Adjusted difference ^ω^
**Energy intake (kJ/day)**			
Baseline, mean (SD)	7,228 (2,343)	7,177 (2,610)	
Follow-up, mean (SD)	6,567 (2,548)	6,496 (2,225)	
Change mean (95% CI) ^†^	−661(−943 to −379) **	−682 (−966 to −397)**	−43 (−386 to 301)
**Fruit g/day**			
Baseline, mean (SD)	189.11 (109.88)	181.56 (115.89)	
Follow up Mean (SD)	193.49 (112.43)	198.01 (118.39)	
Change mean (95% CI)	4.38 (−9.46 to 18.24)	16.45 (2.50 to 30.40)*	9.17 (−9.22 to 27.55)
**Vegetables g/day**			
Baseline mean (SD)	171.71 (76.01)	169.70 (74.27)	
Follow up mean (SD)	173.82 (76.41)	172.79 (65.97)	
Change mean (95% CI)	2.11 (−6.72 to 10.93)	3.09 (−5.3 to 11.53)	0.18 (−11.53 to 11.89)
**Snack food, e.g., cakes/pastries/crisps g/day**			
Baseline mean (SD)	43.66 (45.57)	50.03 (51.63)	
Follow up mean (SD)	34.57 (32.08)	35.33 (30.14)	
Change mean (95% CI)	−9.08 (−14.27 to −3.90)*	−14.69 (−20.54 to −8.84)*	−1.88 (−7.18 to 3.43)
**Takeaway food, e.g., pizza, hamburger g/day**			
Baseline mean (SD)	60.18 (46.09)	61.98 (50.21)	
Follow up mean (SD)	55.41 (50.24)	56.39 (58.00)	
Change mean (95% CI)	−4.76 (−10.38 to 0.86)	−5.58 (−12.16 to −0.99)*	−0.07 (−7.79 to 7.64)
**Bread g/day**			
Baseline mean (SD)	57.64 (36.47)	63.47 (35.05)	
Follow up mean (SD)	54.00 (33.57)	53.69 (32.11)	
Change mean (95% CI)	−3.64 (−8.53 to1.24)	−9.73 (−14.04 to −5.45)*	−3.15 (−8.59 to 2.27)
**Breakfast cereal g/day**			
Baseline mean (SD)	72.61 (72.00)	63.07 (59.98)	
Follow up mean (SD)	69.65 (78.19)	67.68 (83.27)	
Change mean (95% CI)	−2.95 (−11.6 to 5.75)	4.61 (−6.16 to 15.39)	4.30 (−9.27 to 17.87)
**Alcohol g/day**			
Baseline mean (SD)	121.52 (246.53)	122.39 (208.57)	
Follow up mean (SD)	93.26 (170.44)	93.82 (172.30)	
Change mean (95% CI)	−28.25 (−54.20 to −2.31) *	−28.58 (−48.15 to −9.01) *	0.11 (−27.02 to 27.23)
**Leisure time activity (MET-min/week)** ^¥^			
Baseline mean (SD)	863 (1,228)	925 (1,760)	
Follow-up mean (SD)	974 (1,276)	879 (1,128)	
Change mean (95% CI)	111 (−7 to 300)	−46 (−256 to 164)	−118 (−334 to 98)
**Sitting time hrs/day**			
Baseline mean (SD)	3.66 (2.31)	3.91 (2.24)	
Follow-up mean (SD)	3.69 (2.29)	3.84 (2.52)	
Change mean (95% CI)	0.03 (−0.24 to 0.30)	−0.06 (−0.34 to 0.22)	−0.01 (−0.33 to 0.31)
**Diet self-management** ^#^			
Baseline mean (SD)	2.74 (0.65)	2.70 (0.61)	
Follow-up mean (SD)	2.74 (0.60)	2.80 (0.61)	
Change mean (95% CI)	0.00 (−0.06 to 0.07)	0.10 (0.04 to 0.16) *	0.08 (0.00 to 0.17) *
**Activity self-management** ^#^			
Baseline mean (SD)	2.76 (0.74)	2.66 (0.65)	
Follow-up mean (SD)	2.78 (0.71)	2.78 (0.67)	
Change mean (95% CI)	0.02 (−0.05 to 0.11)	0.12 (0.04 to 0.19)*	0.06 (−0.02 to 0.15)

*p < 0.05

** *p* < 0.01

^†^ Indicates paired Student’s *t* tests used to calculate all change scores.

^#^ Self-management is presented as a mean, measured on a Likert scale of 1–5 with higher scores indicating greater self-management strategy use.

^ω^ Linear regression adjusted for baseline values and cluster.

^¥^ Leisure time activity has been calculated using the leisure activity domain only of the IPAQ long version and expressed as MET-min calculated by multiplying the MET activity level (walking 3.3, moderate 4.0 and vigorous 8.0) by minutes performed each day.

### Program Satisfaction

A full community evaluation and economic analysis will be reported elsewhere [24]. In summary, integration with local community organisations (local government, schools, kindergartens, health programs, and community centres) was successful. Local organisations provided recruiting support, venues, and resources, and linked us with compatible local lifestyle programs and stakeholders. Of the intervention participants, 258 (74%) received phone coaching. Of those who did not receive phone coaching, 79 participants were unreachable despite several contact attempts, eight participants withdrew from the study, and two advised they were pregnant and therefore ineligible.

Strategies found to be valuable by intervention subjects were the group session (3.6 on a 5-point Likert scale where 1 = not at all helpful and 5 = extremely helpful), “SMS messages” (3.5), “information about prevention of weight gain” (3.4), “phone coaching” (3.2), “program manual” (3.2), “weighing yourself” (3.2). The least helpful was the “website” (2.1). Most women attended the program for personal health reasons, the most common being, “I did not want to gain more weight,” “I wanted support and motivation to change my lifestyle,” “to help research,” and “to improve my health.” Compared to the control group, the intervention group found the program “assisted them to be more active and eat a healthy diet” (3.2 versus 2.6, *p* < 0.001 on a Likert scale), and they “would recommend the program” (3.6 versus 3.1, *p* < 0.001), with both groups reporting a similar “effort in following the advice given in the program” (2.9 versus 2.8, *p* = 0.15).

## Discussion

In this large, pragmatic RCT, we found that a low intensity community-integrated lifestyle intervention prevented weight gain in rural-dwelling women compared to a control group who continued to gain weight similar to that reported in the background population. We observed an intervention effect on weight gain across all BMI subgroups. Greater weight loss was noted in intervention women who regularly self-weighed, confirming self-weighing is an important behavioural strategy when paired with an intervention. Successful uptake and utilisation of intervention messages was demonstrated by improvements in diet quality and self-management, a core behavioural component of the intervention.

The US Agency for Healthcare Research and Quality defines a between-group difference in weight gain of 0.5 kg as significant and meaningful [9]. The clinical significance of the observed ~1 kg weight difference is underscored by the association between similar weight gain and breast cancer [25], hypertension [26], type 2 diabetes [27], and coronary heart disease in women [28]. Modelling a 1% reduction in BMI (equivalent to approximately 1 kg for an average adult) across the US population, Wang et al. estimated this small weight change would avoid 2 million cases of diabetes, 1.5 million cardiovascular diseases, and over 73,000 cases of cancer [2]. The weight gain reported in the control group (0.44 kg) is less than the self-reported weight gain in young rural Australian women (0.7 kg) [10]. Weight gain in control groups is highly variable [7] and simply participating in data collection and weight measures may motivate behaviour change in some participants [29]. The study was designed to limit this effect by minimising contact, yet a small intervention effect in the control group associated with participation may have led to an underestimation of the true intervention impact.

Despite guidelines advising health care professionals to treat, monitor and prevent weight gain in all BMI categories, few empirical, accessible, and affordable interventions exist. Weight-loss trials are characterised by intensive frequent contact with prescriptive diet and physical activity advice. Such interventions achieve weight loss, although up to 50% of lost weight may be regained by 12 mo [30]. In addition, the high participant burden from comprehensive diary keeping or daily website logs, as well as a lack of personal control evident in intensive trials limits acceptability. This is demonstrated by poor attendance [31], failure to complete homework assignments, and low message uptake with attrition rates up to 50% at 12 mo [32]. In contrast, we demonstrated weight gain prevention is feasible, highly acceptable to participants, and can be achieved with low participant burden [16] and good retention at around 79%.

Identifying the successful elements of interventions targeting multiple behaviours is complex. Elements of behavioural interventions that impact outcomes are reported to be frequent contact, social support, and using recognised behaviour change strategies (e.g., goal-setting) [16]. In contrast, our trial had few contacts but included components valued by participants. These were simple health messages, a focus on small changes to behaviour, and the application of nonprescriptive, self-management behaviour change strategies and ongoing support via SMS text reminders. The ideal number, length, and type of contacts for the primary prevention of weight gain in women is unknown, with previous behaviour-based trials varying in length and contacts. For example, an intervention of 4 group sessions plus text messages over 1 yr in a general population of women achieved a weight difference between groups equal to 1.1 kg [22]. In contrast, a higher intensity intervention of similar duration (12 individual contacts plus gym memberships, phone and voice feedback) achieved a marginally greater weight difference than we observed [33]. In a 2 yr trial, 15 group sessions did not achieve a significant weight difference [34], whilst a 5 yr intervention consisting of 21 group contacts plus 30 reviews showed a between-group weight difference of just 2.3 kg [35]. In comparison, we demonstrated a significant weight difference with less intensity, combining one group and one telephone call, plus monthly text message support. Our self-management approach with activities to enhance competence, personal decision making, and confidence was designed to increase intrinsic motivation shown to be important for sustained behaviour change [36]. Low participant burden and simple messages aimed to increase message uptake, program acceptability and retention. Similar program elements have been used in urban women [22] and in pregnant women [37], and those results confirm the specific set of program elements described here may have contributed to the success of the intervention without the need for frequent intensive contact.

Delivery of lifestyle interventions has changed from predominantly face-to-face to more remote delivery in recent years. The use of phone, mail, and Internet interventions is logical and low cost at the population level, but lacks the social connection known to be important. To date no single mode of delivery such as SMS or web-based offers a clear advantage [38–40]. In contrast, mixed modes of delivery have been shown to enhance behaviour change [41]. By including mixed modes of delivery characterised by face-to-face, phone, SMS text support, and a manual, all of which achieved high participant satisfaction, we were able to address common behavioural barriers including “not getting started,” “forgetting to act,” and “getting derailed,” [29] as well as time and childcare constraints. Here, the facilitated face-to-face session assisted women to formulate intentions and action plans; group delivery stimulated social support; the monthly text message was a reminder to act; and the phone coaching helped retrack behaviours. This is consistent with behavioural theory where people who have formed action plans are likely to be successful compared to merely considering a goal [29]. Overall, our results have implications for the delivery of behavioural lifestyle programs targeting women where a mix of personal and electronic delivery appears to be ideal. This mix offers flexibility for both health care providers and participants where costs can be retained, barriers to delivery and attendance are addressed, and the length of interventions and support can be extended, resulting in greater satisfaction and long-term adherence.

Strengths of the trial include the robust pragmatic design with few exclusion criteria and high external validity. The groups studied were recruited from a general population of women from disadvantaged areas with generally lower socioeconomic status. The pragmatic design and delivery makes the intervention readily scalable. The large scale of the trial along with cluster design and the low intensity delivery targeting prevention of weight gain is novel and are strengths. Limitations include the lack of available sensitive measurement tools to assess the small persistent annual energy deficit required to achieve the observed results (estimated between 100–200 kcals per day [42,43]). Therefore, the physical activity and energy intake data should be interpreted with caution. The loss to follow-up was within the anticipated range and less than other lifestyle interventions [32]. Those who had a higher BMI at baseline or who gained weight during the intervention period may have been less inclined to complete 1 yr weight. However, we were unable to detect a difference in baseline characteristics between those who completed 1 yr weight and those who did not (Table 4). As dropout rates were similar in intervention and control groups, impact on the observed difference in weight would be expected to be negligible. A longer follow-up would assist the assessment of duration of behaviour change in this target group.

### Translation into Policy and Practice

Policy and practice implications are evident with population strategies incorporating targets to halt the rise in obesity prevalence being implemented in England (Healthy Lives, Healthy People), the US (Healthy People 2020) [44], Europe (Gaining Health), and Australia (The Healthiest Country 2020) [45], despite a lack of available evidence-based interventions. The pragmatic trial design we used enables a rapid research to practice implementation cycle. The findings also support clinical application where prevention of weight gain is an attainable and satisfying goal for patients, is feasible to deliver, and requires few resources and health practitioner time. At a population level, the HeLP-her program integrated with a whole population approach can form part of a suite of interventions to address the complex issue of obesity prevention demonstrated by translation activities using the HeLP-her program currently occurring in Australia [46].

### Conclusions

In summary, our results show a low-intensity lifestyle program was able to prevent weight gain in a general population of women. Key features of the intervention included community integration, a focus on small changes to behaviour, self-management, minimal participant burden, and a mix of personal and electronic modes of delivery. The findings advance the limited literature on effective weight gain prevention strategies and align with clinical guidelines and international goals to halt the rise in obesity prevalence.

## Supporting Information

S1 Text CONSORT checklist. (DOCX)

## Abbreviations

BMI: body mass index
FFQ: Food Frequency Questionnaire
IPAQ: international physical activity questionnaire
IQR: Inter Quartile Range
MET-min: metabolic equivalent expressed in minutes of activity per day
RCT: randomised controlled trial
SEIFA: socioeconomic index for areas
VIF: variance inflation factor
